# Anticancer therapy at end-of-life: A retrospective cohort study

**DOI:** 10.2340/1651-226X.2024.22139

**Published:** 2024-05-08

**Authors:** Johnny Singh, Andreas Stensvold A, Martin Turzer, Ellen Karine Grov

**Affiliations:** a Østfold Hospital Trust, Department of Oncology, Graalum, Norway; b Oslo Metropolitan University, Faculty of Health Sciences, Institute of Nursing and Health Promotion, Oslo, Norway

**Keywords:** Neoplasms, end of life care, palliative care, quality of health care, drug therapy

## Abstract

**Background:**

A significant proportion of patients with incurable cancer receive systemic anticancer therapy (SACT) within their last 30 days of life (DOL). The treatment has questionable benefit, nevertheless is considered a quality indicator of end-of-life (EOL) care. This retrospective cohort study aims to investigate the rates and potential predictors of SACT and factors associated with SACT within the last 30 DOL. The study also evaluates the scope of Eastern Cooperative Oncology Group (ECOG) performance status and the modified Glasgow prognostic score (mGPS) as decision-making tools for oncologists.

**Patients and Material:**

This review of medical records included 383 patients with non-curable cancer who died between July 2018 and December 2019. Descriptive statistics with Chi-squared tests and regression analysis were used to identify factors associated with SACT within the last 30 DOL.

**Results:**

Fifty-seven (15%) patients received SACT within the last 30 DOL. SACT within 30 last DOL was associated with shorter time from diagnosis until death (median 234 days vs. 482, *p* = 0.008) and ECOG score < 3 30 days prior to death (*p* = 0.001). Patients receiving SACT during the last 30 DOL were more likely to be hospitalised and die in hospital. ECOG and mGPS score were stated at start last line of treatment only in 139 (51%) and 135 (49%) respectively.

**Interpretation:**

Those with short time since diagnosis tended to receive SACT more frequently the last 30 DOL. The use of mGPS as a decision-making tool is modest, and there is lack in documentation of performance status.

## Background

The therapeutic landscape in medical oncology has changed rapidly in the last decade and continues to evolve. Systemic anticancer treatment (SACT) now includes immune checkpoint inhibitors (ICI), targeted agents and anti-hormonal treatment, alongside chemotherapy [1]. Combinations of treatment modalities are also being explored with promising prospects [2, 3]. The aim of treatment and follow-up among cancer patients in the palliative phase is to prolong survival, improve quality of life (QoL), and achieve symptom relief [4].

However, chemotherapy to cancer patients in the palliative phase, particularly in the last 30 days of life (DOL) might have a negative impact on QoL [5]. It may also lead to increased rate of death at intensive care units (ICU), death at non-preferable locations, and it is time consuming for the patient [5, 6]. Check point inhibitors and targeted therapy are in general associated with lower risk of adverse side effects compared to chemotherapy, but though not negligible [7, 8]. Immune checkpoint inhibitors also offer the possibility of a complete response (absence of detectable disease). Unfortunately, time to response is long and patients may not live long enough for the response to emerge. The absence of biomarkers can also make it hard to predict response.

Earle et al. [9] proposed three major concepts as quality indicators for end-of-life (EOL) care: introduction of new anti-cancer therapies or continuation of ongoing treatment at EOL, repeated hospital admissions or ICU admissions, and late or no referral to palliative care. The European Society of Medical Oncology (ESMO) also advises against the use of chemotherapy, immuno-therapy and radiotherapy at EOL [10]. The rate of SACT in the last 30 DOL among patients in the palliative phase varies considerable between 4 and >50%, with higher rates in young patients and those with higher socioeconomic status [11–13]. In the recent years, there seems to have been a decrease in the use of chemotherapy with a concomitant increase in use of ICIs at EOL [14].

The shared decision-making (SDM) process regarding starting and ceasing SACT at EOL is therefore an ongoing challenge for both health personnel, patients and family caregivers because all parts have to decide based on, for them, relevant information. The introduction of the new anticancer agents has made the ethical and clinical considerations even harder because neither benefit nor toxicity is guaranteed. There is also a raising concern regarding the heavy financial burden on the health care system contributed by futile treatment at EOL.

Making decisions concerning treatment and EOL wishes in collaboration between health personnel, patients and sometimes the patient’s caregivers has a democratic aspect as it highlights the perspectives of the involved [15]. Leaving the patient with a sense of control and partnership in the decision-making process [16] might result in a shared process. However, decisions must be taken from knowledge about the treatment’s possible consequences and side-effects, and thereby follows acknowledgement of valuable information from health personnel. In this study, we shed light on this perspective and have the focus on the last 30 DOL and medical aspects in this short trajectory.

Prognostication is a difficult task and even experienced oncologists can over- or underestimate survival among patients in the palliative phase [17]. Validated prognostic scales can be helpful when considering starting and ceasing SACT, and Eastern Cooperative Oncology Group performance status (ECOG) is a strong prognostic factor [18]. Glasgow prognostic score (GPS) and the modified version, modified Glasgow Prognostic Score (mGPS) (based on CRP- and albumin-values) are also of prognostic value independently of performance status [19–22]. International guidelines advice against the use of chemotherapy at ECOG ≥ 3, based on the evidenced based association between reduced performance status and reduced survival, treatment response, and increased risk of treatment toxicity [23]. The amount of and associated factors such as performance status, QoL and GPS in connection with the use of check point inhibitors and targeted therapy at EOL is however, still sparsely documented. There are indications that ICI does not overcome the negative prognostic role of poor performance status and that ICI use in the last 30 DOL is associated with hospital deaths and repeated admissions [24–26].

The overall aim of this study was therefore to investigate the rates, potential predictors and associated factors regarding SACT within the last 30 DOL at a Cancer Department in a local hospital. Furthermore, we wanted to evaluate the scope of ECOG and mGPS as decision-making tools, and map the use of radiotherapy within the last 30 DOL.

## Methods

### Ethics approval

The study was evaluated and approved by the Data Protection Official at the hospital. The Regional Committee of Ethics in Norway evaluated the study and found it not to be within the mandate of the Norwegian Health legislation (# 593639, 05/15/2023). Informed consent was waived after assessment and approval by the Data Protection Official at The Østfold Hospital Trust (Data Protection Official, dated 13.02.2020). The study was carried out in accordance with relevant guidelines and regulations. The STROBE guidelines were utilised.

## Material

We conducted a retrospective review of medical records for all patients who died between the pre-Covid period July 1st 2018 and December 31st 2019 and were treated at the Oncology Department. Patients were eligible for the study if the malignant disease was documented in the patient record to be incurable and non-haematological. Since paediatric patients and patients with primary gynaecologic cancer, head and neck cancer, pulmonary and neuro malignancies were treated in other departments, we did not include those. When information on ECOG was missing, performance status was attempted estimated from other information in the medical record by the first author (JS). Data on age, gender, cancer type, treatment lines, hospitalisations and health care services utilisations, use of palliative unit, hospital deaths, SACT and radiotherapy utilisation were collected.

### Modified Glasgow Prognostic Score

CRP and albumin were registered if the blood sample was taken within 14 days prior of the date of the decision to start last line of SACT and/or 30–44 days prior to death.

CRP > 10 mg/ml and albumin < 35 g/l gives a score of 2, CRP > 10 mg/ml and albumin > 35 g/l gives a score 1, and CRP < 10 mg/ml scores 0 independently of albumin level. A score of 1 or 2 is associated with a poor prognosis [19].

### Statistical considerations

Dichotomisation was performed based on the presence or absence of use of SACT within the last 30 DOL. For categorical variables, Pearson’s chi-squared test was used for group comparisons. Cox regression analysis and Kaplan-Meier test were used for exploring significance and differences in survival time. To analyse which factors were most significantly associated with SACT at EOL, we used logistic regression analysis. Multiple imputation was used for ECOG at start last line of treatment and 30 days prior to death where approximately 30% of the data were missing. Due to lack of data in over 50% of the cases, mGPS was not eligible for multiple imputation and therefore excluded from the regression analysis. All patients had a cancer diagnosis; however, particular cancer type was excluded from the regressions analysis due to low frequency in many of the cancer types. Significance level was defined as < 0.05 and all testes were two-tailed.

## Results

**Figure 1:**
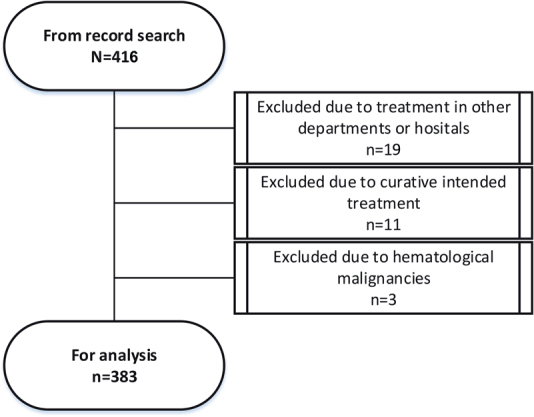
Flow chart of the sample.

In total, 416 patients were identified from record search and among these 383 patients were analysed. Reasons for exclusion are given in Figure 1.

A total of 237 (62%) of the included patients were men. Mean age at death was 71 years. Median survival from diagnosis of incurable disease to death was 295 days. A total of 275 patients (72%) received SACT. Characteristics of patients and intensiveness of care among patients receiving no SACT within 30 DOL and receiving SACT other than hormone treatment within 30 DOL is found in Table 1. Among the 116 (30%) patients who received SACT within 30 last DOL, 59 (15%) received anti-hormonal treatment alone, 27 (7%) received chemotherapy alone, 15 (4%) received targeted therapy, and 4 (1%) received check point inhibitors. In addition, 6 patients (2%) received both targeted therapy and anti-hormonal treatment, 3 (1%) both chemotherapy and targeted therapy, and 2 (1%) chemotherapy and anti-hormonal treatment. A total of 272 patients (71%) were hospitalised within the last 30 DOL. Patients receiving SACT during the last 30 DOL were more likely to be admitted to hospital within the last 30 DOL (91% vs. 67%, *p* < 0.001) and to a higher degree tended to die in hospital (63% vs. 20%, *p* < 0.001). Of the 275 patients receiving SACT, 61 (22%) were referred to palliative care unit (PCU) by start last line of treatment.

**Table 1:** Characteristics of patients and intensiveness of care among patients receiving no SACT within the 30 last days of life and receiving SACT within the 30 last days of life. The p-values are for comparisons with the group who did not receive SACT.

Demographic and clinical variables	SACT* during 30 last days, *n* (%)		*p-value*
	Yes *n* = 57	No *n* = 326	
Gender Male Female	35 (61%) 22 (39%)	202 (61%) 124 (38%)	0.94
Age at death Mean (years) ≥ 70 60–69 < 60	66 27 (47%) 12 (21%) 18 (32%)	72 212 (65%) 69 (21%) 45 (14%)	0.002 0.01 1.00 **< 0.001**
Time from diagnosis until death (mean days)	637.5 ± 120.4	713.7 ± 60.0	0.54
Patients with minor children	12 (21%)	17 (5%)	**< 0.001**
Multiple primary malignancies	1 (2%)	22 (7%)	0.14
Treatment initiated at a different hospital	3 (5%)	20 (6%)	0.80
Cancer type Esophageal Ventricular Hepatic Bile duct Pancreatic Colorectal Kidney Urothelial Breast Prostate Malignant melanoma Cancer of unknown primary cite Others	3 (5%) 4 (7%) 1 (2%) 1 (1%) 7 (12%) 7 (12%)¹ 6 (11%) 2 (4%) 11 (19%)² 8 (14%) 7 (12%) 0 (0%) 0 (0%)	13 (3%) 13 (4%) 7 (2%) 21 (6%) 48 (15%) 75 (23%) 18 (6%) 21(6%) 30 (9%) 46 (14%) 11 (3%) 11 (3%) 12 (4%)	
Line of treatment Never treated First line Second line Third line Fourth line or greater	22 (39%) 12 (21%) 6 (11%) 17 (30%)	108 (33%) 95 (29%) 60 (18%) 26 (8%) 37 (11%)	0.15 0.64 0.52 **< 0.001**
Number of hospitalisations within last 30 days None 1 2 ≥ 3	5 (9%) 33 (56%) 17 (30%) 3 (5%)	106 (33%) 147 (45%) 58 (18%) 15 (5%)	**< 0.001** 0.07 **0.03** 0.83
Hospitalisation > 7 days within last 30 days Yes No	18 (32%)³ 39 (68%)	80 (25%) 246 (75%)	0.26
Use of palliative unit within last 30 days Yes No	25 (44%) 32 (56%)⁴	176 (54%) 150 (46%)	0.16
ICU admission within last 30 days Yes No	2 (4%) 55 (96%)	4 (1%) 322 (99%)	0.20
Major surgery within last 30 days Yes No	1 (2%) 56 (98%)	2 (1%) 324 (99%)	0.37
Invasive nutrition support within last 30 days Yes No	5 (9%)⁵ 52 (91%)	38 (12%)⁶ 288 (88%)	0.52
Erythrocyte transfusion within last 30 days Yes No	14 (25%) 43 (75%)	77 (24%) 249 (76%)	0.88
Demographic and clinical variables	SACT* during 30 last days, *n* (%)	*p-value*	
	Yes *n* = 57	No *n* = 326	
Hospital death Yes No	36 (63%) 21 (37%)	66 (20%) 260 (80%)	**< 0.001**

SACT: systemic anticancer therapy.

* Anti-hormonal treatment included as treatment line, but not as SACT during 30 last days.

¹ One patient received both chemotherapy and targeted therapy.

² One patient received both chemotherapy and targeted therapy.

³ One patient received both chemotherapy and targeted therapy.

⁴ One patient received both chemotherapy and targeted therapy.

⁵ All five patient received parenteral nutrition support.

⁶ Twenty nine patients received parenteral nutrition, eight received enteral tube nutrition, and one received both.

Among patients receiving SACT other than anti-hormonal treatment within the last 30 DOL (57), 22 (39%) received only one line of treatment. Characterisation of these patients is found in Table 2.

**Table 2:** Characterisation of patients receiving SACT other than anti-hormonal treatment within last 30 DOL and only one line of treatment.

Factor	*n* = 22
Cancer type Colorectal¹ Ventricular Pancreatic Malignant melanoma Kidney Esophageal Bile duct Breast Urothelial	6 3 3 3 3 1 1 1 1
Gender Male Female	14 8
Age at death (median years)	69
Time from diagnosis until death (median days)	75
Time from start last line of treatment until death (median days)	53
ECOG start last line of treatment 0 1 2 3 Not stated	4 5 5 0 8
ECOG 30 days prior to death 0 1 2 3 Not stated	3 4 6 1 8
mGPS start last line of treatment 0 1 2 No information	5 6 5 6
mGPS 30 days prior to death 0 1 2 No information	2 4 8 8
Referred to PCU by start last line of treatment Yes No	4 18
Contact with PCU within the last 30 DOL Yes No	7 15

ECOG: Eastern Cooperative Oncology Group; mGPS: modified Glasgow prognostic score; PCU: palliative care unit; DOL: days of life.

¹ One patient received both chemotherapy and targeted therapy.

**Figure 2:**
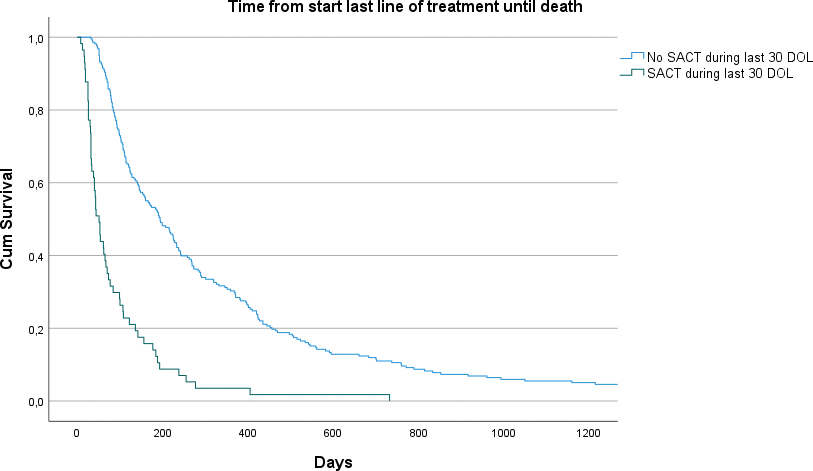
Kaplan-Meier graph of time from start last line of treatment until death.

Median survival from start of last line treatment among patients receiving SACT was 51 days (CI: 38.3 – 63.7) compared to 194 days (CI: 153.1 – 234.9) among patients not receiving SACT within the last 30 DOL (*p* < 0.001, Figure 2).

**Figure 3:**
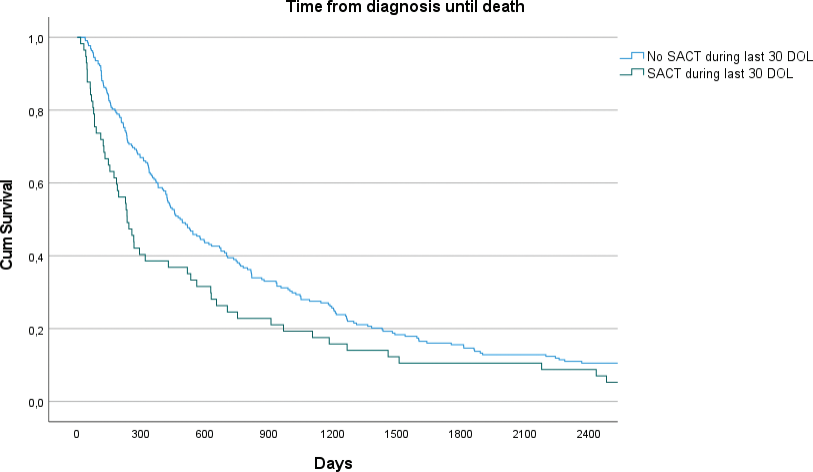
Kaplan-Meier graph of time from diagnosis until death among patients receiving SACT.

Median survival from diagnosis among patients receiving SACT within the last 30 DOL was 234 days (CI: 159.0 – 309.0) compared to 482 days (CI: 382.0–582.0) among patients receiving SACT, but not within the last 30 DOL (*p* = 0.008, Figure 3).

Radiotherapy was given to 18 (5%) patients within the last 30 DOL. Spinal cord compression was the most common indication (6 patients). Only 13 patients completed radiotherapy as scheduled (Table 3). There was no association between radiotherapy within 30 DOL and SACT, as only one patient received both targeted therapy and radiotherapy.

**Table 3:** Eighteen patients (5%) received radiotherapy within 30 last DOL.

	*n* = 18
Cancer type Prostate Malignant melanoma Ventricular Pancreatic Colorectal Urothelial Bile duct Others	4 4 2 2 2 2 1 1
Indication Spinal cord compression Brain metastasis Painful bone metastasis Painful soft tissue metastasis Bleeding tumour	8 4 3 2 1
Fractionation schedules 1–2 3–5 6–10	4 8 6
Completed radiotherapy as planned	13
Causes for not completing radiotherapy as planned Poor performance status Progressive disease Septicaemia Death	2 1 1 1

Cancer types, indications, fraction schedules, completion rate and reasons for discontinuation of radiotherapy within 30 last DOL are listed.

Eastern Cooperative Oncology Group was stated at start last line of treatment in 139 patients (51%). Performance status was estimated in additional 50 patients. In 86 (31%) patients, information in the patients’ medical records was not sufficient for such estimation due to lack of documentation. Systemic anticancer treatment during the last 30 DOL and connected performance status and mGPS are found in Table 4.

**Table 4:** Performance status and modified Glasgow prognostic score among patients who received at least one line of SACT.

	SACT* during 30 last days, *n* (%)		*p*
	Yes *n* = 57	No *n* = 218	
Performance status at start last line of treatment ECOG 0 ECOG 1 ECOG 2 ECOG ≥3 No information	6 (11%) 20 (35%) 17 (30%) 3 (5%)¹ 11 (19%)	17 (8%) 57 (26%) 56 (26%) 13 (6%) 75 (34%)	0.51 0.18 0.53 0.84 0.03
Performance status 30 days prior to death ECOG 0 ECOG 1 ECOG 2 ECOG ≥3 No information	5 (9%) 13 (23%) 18 (32%) 8 (14%) 13 (23%)	0 (< 1%) 13 (6%) 21 (10%) 114 (52%) 70 (32%)	< 0.001 < 0.001 < 0.001 < 0.001 0.17
mGlasgow prognostic score at start last line of treatment 0 1 2 No data	11 (19%) 9 (16%) 15 (26%) 22 (39%)	31 (14%) 39 (18%) 35 (16%) 113 (52%)	0.35 0.71 0.07 0.08
mGlasgow prognostic score 30 days prior to death 0 1 2 No data	4 (7%) 8 (14%)² 17 (30%)³ 28 (49%)	10 (5%) 10 (5%) 71 (33%) 127 (58%)	0.46 0.01 0.69 0.22

SACT: systemic anticancer therapy; ECOG: Eastern Cooperative Oncology Group.

* Anti-hormonal treatment included as treatment line, but not as SACT during 30 last days.

¹ Two received targeted therapy and one ICI.

² One patient receiving both chemotherapy and targeted therapy.

³ One patient receiving both chemotherapy and targeted therapy.

Only ECOG < 3 30 days prior to death remained associated with SACT within the last 30 DOL in the regression analysis (*p* < 0.001) among patients receiving at least one line of SACT. Age < 70, < 3 treatment lines, and SACT given within the last 30 DOL were associated with shorter survival from diagnosis among patients receiving at least one line of SACT (Table 5).

**Table 5:** Regression analysis for the impact of demographic and clinical variables on survival from diagnosis until death among patients receiving at least one line of SACT.

Covariates	Standard error	*P*	HR	95% CI¹	
				Lower	Upper
Age 60 60–69 ≥ 70*	0.170 0.155	**< 0.001** **0.043**	2.351 1.369	1.685 1.010	3.280 1.857
Gender Male Female*	0.132	0.310	1.143	0.883	1.479
SACT during last 30 DOL Yes No*	0.161	**< 0.001**	2.287	1.668	3.134
Treatment lines < 3 ≥ 3*	0.145	**< 0.001**	3.443	2.593	4.570

CI: confidence interval; HR: Hazard ratio; SACT: systemic anticancer therapy; DOL: days of life

* Reference group.

¹ 95% CI for Hazard ratio.

## Discussion

In this cohort, we wanted to investigate the rates, potential predictors and associated factors regarding SACT at EOL. We also wanted to investigate the use of ECOG and mGPS as decision-making tools. A total of 15% patients received SACT the last 30 DOL. By excluding the 108 (28%) included patients never receiving any SACT at all, the proportion of patients receiving SACT the last 30 DOL might be regarded higher at 21%. Only ECOG < 3 30 days prior to death remained associated with SACT within the last 30 DOL in the regression analysis. Patients receiving SACT during the last 30 DOL were more likely to be admitted to hospital within 30 last DOL and die in hospital. Eastern Cooperative Oncology Group and mGPS were stated at start last line of treatment in only half of the patients.

The use of SACT within the last 30 DOL in this cohort was comparable to findings in other recent international reports [12, 14, 27, 28]. The findings are however, not necessarily easily comparable. Studies conducted in different countries may have variations in patient selection, healthcare organisation, and economic incentives. A recent domestic nationwide register study reported rate at only 3% [29]. The reason for this discrepancy with our findings is uncertain. It may be attributed to the limited number of patients included in our study or regional differences in treatment tradition and attitude towards EOL treatment. The frequency of immunotherapy treatment at EOL was lower compared to findings indicating increased frequency [30]. However, the limited number of patients in our cohort makes conclusions uncertain.

Timeline from diagnosis until death was twice as long in the group not receiving SACT within the last 30 DOL. This may be due to higher acceptance of limitations in treatment utility among oncologist and patients in later course of the disease. The same is suggested for time from the start of last line of treatment until death, which was longer in the group not receiving SACT within the last 30 DOL. However, we do not know the cause of death among these patients nor deaths attributed to SACT. The fact that cancer patients can get myriad of symptoms, makes it difficult to explain whether patients die due to anticancer therapy toxicity or of other reasons. There are however, indications that anticancer therapy related deaths make up a significant share in cancer patients [31].

The use of mGPS at start of last line of treatment was sparse. This may indicate that mGPS has a modest role as a decision-making tool among oncologists in this study. The low proportion of documented performance statuses indicates a potential for improvement regarding medical record keeping. However, it can also reflect that decision-making processes rest on experiential knowledge and SDM rather than guidelines alone. Significantly better performance status 30 days prior to death among patients receiving SACT within the last 30 DOL, may indicate that these patients were considered to be fit for SACT. However, we do not know if these patients had a subsequently rapid decline in performance status the last 30 DOL due to cancer progression or if their deaths can be attributed to the anticancer therapy toxicity. There can be occasions where both short, predicted prognosis and low performance status may not deter initiation of SACT. Treatment naïve patients with highly treatment sensitive tumours, such as small cell lung cancer, can initially benefit from treatment in terms of response and symptom improvement rates [32]. In our cohort, however, half of the patients who received SACT within the last 30 DOL as their first line of treatment, had gastrointestinal cancers where remarkable response is not the norm.

The use of radiotherapy within the last 30 DOL in our cohort was similar to other reports [33, 34]. Singe fraction radiotherapy (SFRT) utilisation ranged from 0 to 59% in a systematic review [34]. The same review also found a rate of not completing RT of 53–82%, which is higher than our findings at 28%. An explanation can be the small number of patients in our cohort (*n* = 18), which makes interpretation uncertain. We do not neither know if the travel distance to the nearest radiotherapy centre (70 min one way from the hospital) can be a barrier to RT adherence. The high rate of hospitalisation among this group might partly be explained by the travel distance to the nearest RT centre. However, the utilisation of single fraction RT can be argued to be low in this cohort. Several studies indicate that a single fraction RT is just as effective as series of RT fractions when it comes to symptom control, duration of relief and quality of life in patients with bone metastases [35–37]. Single fraction RT has also shown similar outcomes for motor response, bladder function and overall survival in the setting of malignant cord compression in patients with limited prognosis [38]. Additionally, short course whole brain RT for brain metastasis seems to be just as effective in regard to survival and symptom control in this patient group, compared to longer RT courses [39].

Both the American Society of Clinical Oncology (ASCO) and the ESMO advocate the concurrent use of SACT and early involvement of palliative care services [40, 41]. Early palliative care is associated with benefits including better quality of life, less invasive care, and reduced cost at end of life [42–44]. The low proportion of patients referred to the palliative unit indicates a potential for improvement in integrating anticancer therapy with tailored patient-centred supportive care.

### Limitations

Due to limitations in the data set we were not able to assess other factors influencing the use of SACT. Data on comorbidity, symptom burden, rate of symptom relief during EOL, whether treatment was initiated according to national guidelines and patients’ EOL goals were not examined. These factors may influence the decision-making process regarding starting and ceasing SACT. Patients with suspected or confirmed incurable cancer diagnosis never refereed to the Cancer Department, were not included. Many of those may be regarded as obviously not eligible for SACT and therefore not referred to an oncologist. This may represent a potential selection bias to our cohort. A majority of male patients in our study can partly be explained by the exclusion of patients with gynaecologic cancers. The estimation of performance status when missing, was conducted by the first author solely which increases the risk of misclassification bias. Regardless, this study can provide stimuli for further research towards a patient-centred multidisciplinary care, particularly information that gains SDM. This includes more emphasis on the patients’ and caregivers’ perspective, as well as tools for health personnel to lean on, for example, development of improved survival prognostication tools and new predictive tools for anticancer therapy response in the immunotherapy-era.

## Conclusion

The rates of SACT within the last 30 DOL in this cohort are comparable to findings in other recent studies. The use of mGPS as a decision-making tool is modest, and there is a lack in documentation of performance status. Implementation of existing and development of new validated prognostic and predictive tools might be of benefit in integrating anticancer therapy with tailored patient-centred supportive care.

## Declarations

### Availability of data and materials

The data that support the findings of this study are available from the corresponding authors, upon reasonable request.
